# Letter to the Editor: comment on “Association of varicose veins with incidence risk of atrial fibrillation: a population-based cohort study”

**DOI:** 10.1097/JS9.0000000000002157

**Published:** 2024-12-20

**Authors:** Min Wang, Lei Wang, Xingchen Wang, Hongshuo Shi, Huimin Lu

**Affiliations:** a Shandong University of Traditional Chinese Medicine, Jinan, Shandong, China; b The Second Affiliated Hospital of Shandong University of Traditional Chinese Medicine, Jinan, Shandong, People’s Republic of China; c Department of Vascular Surgery, Shuguang Hospital Affiliated to Shanghai University of Traditional Chinese Medicine, Shanghai, China

*Dear Editor,*

I would like to commend the authors on their compelling study, “Association of Varicose Veins with Incidence Risk of Atrial Fibrillation: A Population-Based Cohort Study”^[1]^, which presents valuable insights into a relatively unexplored area of research. The association between varicose veins (VV) and atrial fibrillation (AF) adds a new dimension to understanding cardiovascular risk factors. However, despite the study’s strengths, such as its large sample size and application of propensity score matching (PSM), there are several points worth further exploration and discussion to strengthen future research in this domain.

First, while the authors successfully demonstrate a significant association between VV and AF, they stop short of establishing clear mechanistic pathways or causality. The shared pathophysiology explanation they provide, particularly the role of matrix metalloproteinases (MMPs) in both conditions, is intriguing but underdeveloped. The authors point to MMPs contributing to venous wall remodeling and chronic inflammation, which could link to myocardial remodeling in AF. However, a deeper exploration of this shared pathophysiology through direct measurements of MMP activity or related biomarkers, such as C-reactive protein (CRP) and interleukin-6 (IL-6), would have added substantial value^[2]^. Furthermore, correlating these biomarkers with AF occurrence in patients with VV could establish a more concrete biological pathway. Future research should include a prospective study design that tracks these biomarkers over time in patients with VV to better understand the progression toward AF.

Second, the absence of a significant association between VV and AF in younger individuals raises an important question regarding age-related factors. The authors focus on elderly populations and those with hypertension, which are recognized risk groups for both conditions. However, given the widespread prevalence of VV among younger individuals, it is puzzling that no significant correlation was found in this subgroup. This could indicate that in younger populations, additional factors such as genetic predispositions, lifestyle behaviors (e.g., physical activity levels or occupational factors), or even venous insufficiency at a more subclinical stage may play a role in preventing AF development. A more granular age-stratified analysis and investigation of these other factors could shed light on why the association appears to be weaker in younger groups. Additionally, a study examining the onset of VV symptoms and their duration could clarify whether younger individuals simply have not experienced VV long enough to develop AF, highlighting the importance of longitudinal follow-up in future research.

Third, while the authors acknowledge the limitations of using insurance claims data, this issue requires further elaboration. Claims data can obscure important clinical subtleties, such as the exact clinical stage of VV, as indicated by the CEAP classification. Given that VV severity likely varies significantly among patients, failing to capture this variation could underestimate the true association with AF. Stratifying the cohort based on CEAP classification, especially regarding more severe stages (C4-C6), could reveal a stronger association in patients with advanced VV. Additionally, because claims data do not capture lifestyle or non-pharmacological interventions that might affect outcomes (e.g., the use of compression stockings, exercise regimens), these factors should be included in future studies for more accurate modeling.

The limitations surrounding surgical interventions for VV also merit deeper discussion. The authors mention that insurance claims data did not include all surgical treatments (such as endovenous laser therapy or transilluminated powered phlebectomy), potentially leading to an underestimation of surgical intervention rates. This exclusion leaves an important gap in understanding whether treating VV can mitigate the risk of AF. Furthermore, their conclusion that VV treatments did not reduce AF risk could be skewed by these data omissions. To properly evaluate the effect of VV treatments on AF, future studies should incorporate comprehensive procedural data and stratify patients based on the type and success of surgical interventions. A randomized controlled trial (RCT) assessing AF incidence post-VV treatment would provide much-needed evidence on this front^[3]^.

Finally, the study lacks a comprehensive exploration of confounding factors. Although the authors adjusted for common cardiovascular risk factors, the absence of data on venous thromboembolism (VTE) is concerning, as VTE shares pathophysiological features with both VV and AF. Including VTE as a covariate in future studies could clarify whether its presence is a mediator between VV and AF or simply a shared comorbidity^[4]^.

In conclusion, while this study represents an important step forward, there remain several unanswered questions. Future research should delve deeper into the biological mechanisms linking VV and AF, explore potential protective factors in younger populations, and assess the impact of surgical treatments on reducing AF incidence. Addressing these gaps will ultimately provide a more comprehensive understanding of the relationship between VV and AF, potentially informing more targeted interventions.

## Data Availability

Not applicable.
